# Supplementary material for: Alterations of oral microbiota and impact on the gut microbiome in type 1 diabetes mellitus revealed by integrated multi-omic analyses
Source: Microbiome. 2022 Dec 28;10:243. doi: 10.1186/s40168-022-01435-4 (PMC9795701; doi:10.1186/s40168-022-01435-4)
Supplement: Supplementary file 4 — Additional file 3: Supplementary Figure 1. Oral and gut community structure analysis. Box plots of species richness (a) and alpha diversity (b) between controls and T1DM patients. Hierarchical clustering based on Bray-Curtis distance (c). NMDS ordination based on Bray-Curtis distance (d). All analyses are based on metagenomic data. Supplementary Figure 2. Correlation network of gene transcripts from all samples and differentially active taxa in the oral cavity. The figure corresponds to a subset of the correlation analysis where only the differentially abundant taxa and correlation over 0.7 are plotted. Green and red nodes indicate if the taxon is up- or down-regulated in the oral cavity. Annotations are based on the Pfam database. Supplementary Figure 3. Metaproteomic differences in T1DM at the visit level. Heatmap displaying the relative abundances of human proteins at the visit level with the highest significance in a differential analysis of T1DM. The samples are ordered by conditions. Healthy individuals and T1DM patients are respectively shown in orange and blue boxes. Supplementary Figure 4. Correlations between the abundances of transferred taxa in comparison to their abundance in the oral cavity. The figure shows the correlation between the transfer and the oral cavity. The labels with MG and MT correspond to the abundances at the metagenomic and metatranscriptomic levels for the MG-MT supported variants. MG_only is used for the MG abundances of variants supported by MG reads only and not on the MT level. Colored squares indicate a positive (blue) or negative (red) significant correlations (pval < 0.05). White squares indicate non-significant correlations. Supplementary Figure 5. Correlation analyses of transferred taxa and metadata. The figure shows the correlation between the transfer and the available metadata. The labels with MG and MT correspond to the abundances at the metagenomic and metatranscriptomic levels for the MG-MT supported variants. MG_only [file 40168_2022_1435_MOESM3_ESM.pdf]

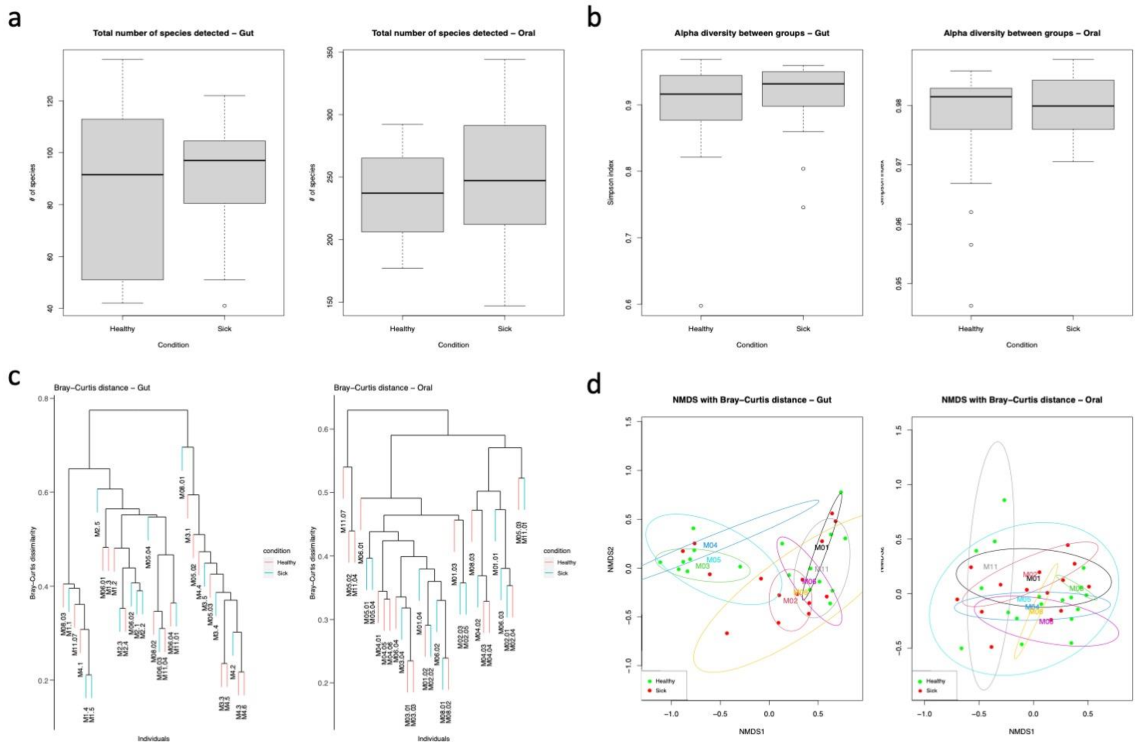

Supplementary Figure 1. Oral and gut community structure analysis. Box plots of species richness (**a**) and alpha diversity (**b**) between controls and T1DM patients. Hierarchical clustering based on Bray-Curtis distance (**c**). NMDS ordination based on Bray-Curtis distance (**d**). All analyses are based on metagenomic data.

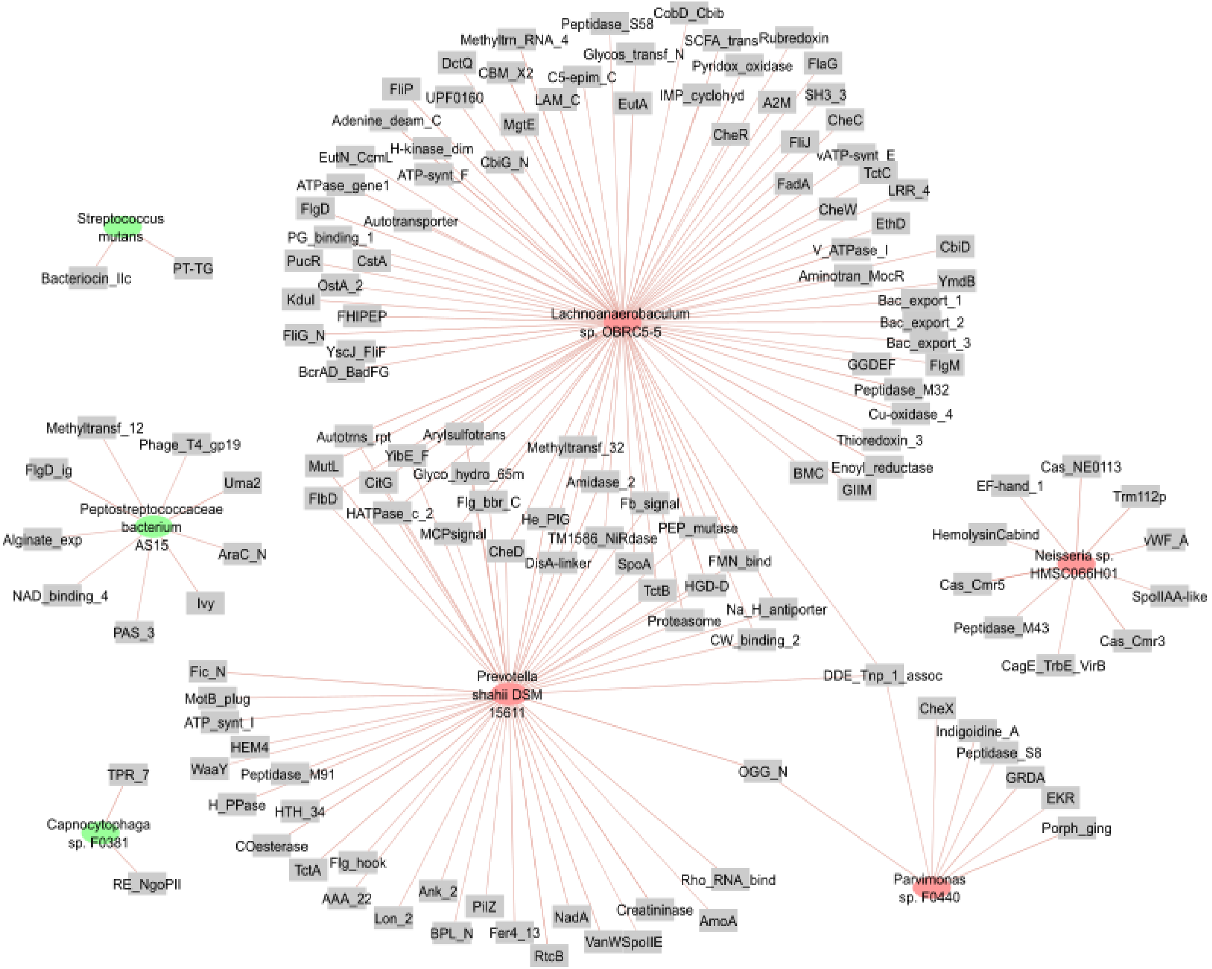

Supplementary Figure 2. Correlation network of gene transcripts from all samples and differentially active taxa in the oral cavity. The figure corresponds to a subset of the correlation analysis where only the differentially abundant taxa and correlation over 0.7 are plotted. Green and red nodes indicate if the taxon is up- or down-regulated in the oral cavity. Annotations are based on the Pfam database.

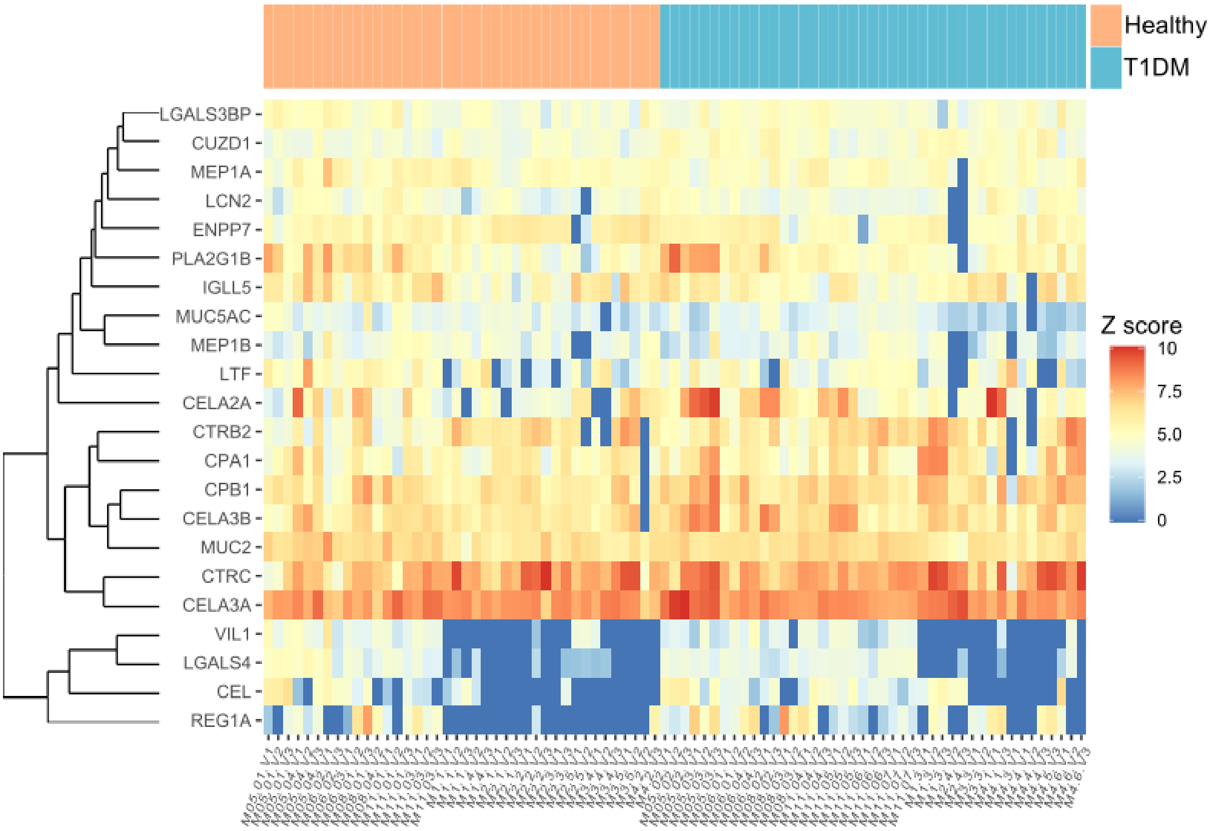

Supplementary Figure 3. Metaproteomic differences in T1DM at the visit level. Heatmap displaying the relative abundances of human proteins at the visit level with the highest significance in a differential analysis of T1DM. The samples are ordered by conditions. Healthy individuals and T1DM patients are respectively shown in orange and blue boxes.

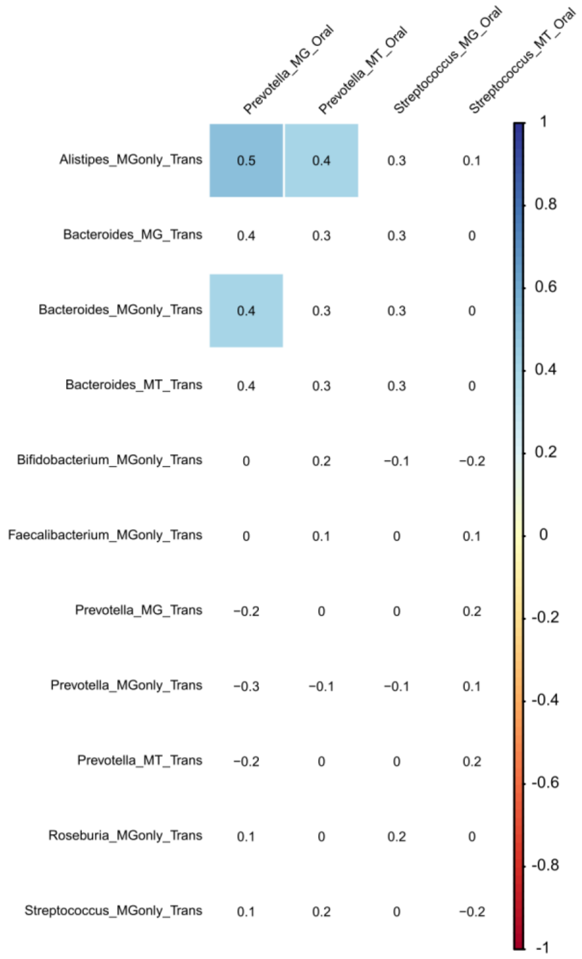

Supplementary Figure 4. Correlations between the abundances of transferred taxa in comparison to their abundance in the oral cavity. The figure shows the correlation between the transfer and the oral cavity. The labels with MG and MT correspond to the abundances at the metagenomic and metatranscriptomic levels for the MG-MT supported variants. MG_only is used for the MG abundances of variants supported by MG reads only and not on the MT level. Colored squares indicate a positive (blue) or negative (red) significant correlations (p-val < 0.05). White squares indicate non-significant correlations.

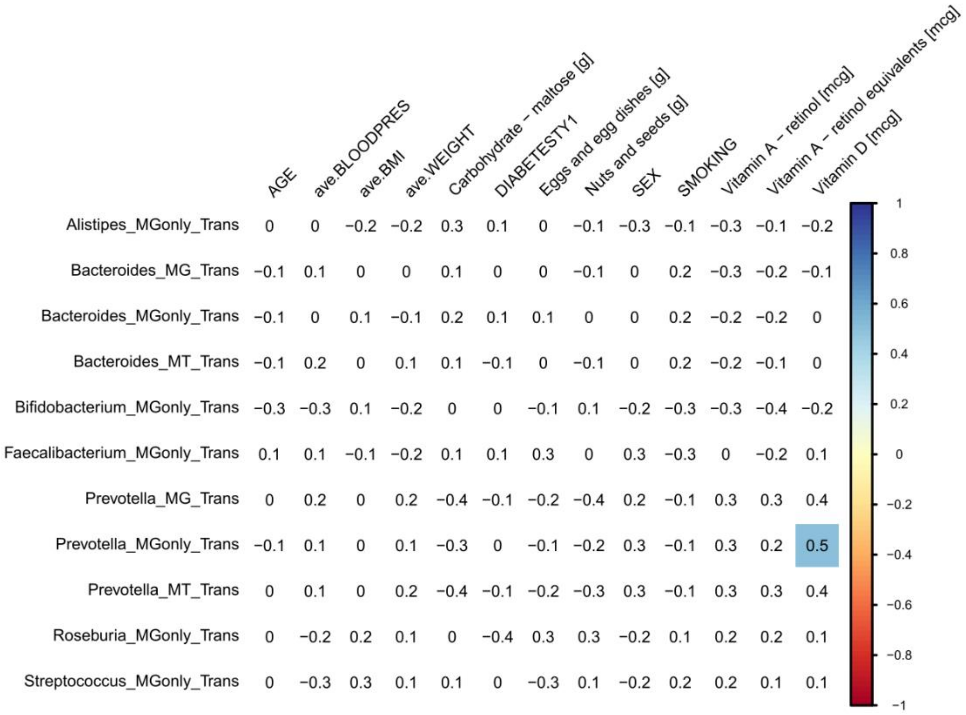

Supplementary Figure 5. Correlation analyses of transferred taxa and metadata. The figure shows the correlation between the transfer and the available metadata. The labels with MG and MT correspond to the abundances at the metagenomic and metatranscriptomic levels for the MG-MT supported variants. MG_only is used for the MG abundances of variants supported by MG reads only and not on the MT level. Colored squares indicate a positive (blue) or negative (red) significant correlations (p-val < 0.05). White squares indicate non-significant correlations.
